# Exploring the smart wearable payment device adoption intention: Using the symmetrical and asymmetrical analysis methods

**DOI:** 10.3389/fpsyg.2022.863544

**Published:** 2022-09-06

**Authors:** Naeem Hayat, Abdullah Al Mamun, Anas A. Salameh, Mohd Helmi Ali, Wan Mohd Hirwani Wan Hussain, Noor Raihani Zainol

**Affiliations:** ^1^Global Entrepreneurship Research and Innovation Centre, Universiti Malaysia Kelantan, Kota Bharu, Malaysia; ^2^UKM – Graduate School of Business, Universiti Kebangsaan Malaysia (National University of Malaysia), Bangi, Malaysia; ^3^College of Business Administration, Prince Sattam Bin Abdulaziz University, Al-Kharj, Saudi Arabia; ^4^Faculty of Entrepreneurship and Business, Universiti Malaysia Kelantan, Kota Bharu, Malaysia

**Keywords:** adoption intention, wearable device, UTAUT, FinTech, Malaysia

## Abstract

The smart wearable device is a new breed of mobile device that offers diversified utilities for health, sport, and finance for consumers worldwide. The current study aims to investigate the provocation of the intention to use smart wearable payment devices among Malaysian consumers. The unified theory of technology acceptance and use of technology (UTAUT) was employed with the cross-sectional survey-based data to explain the adoption of the smart wearable payment device. Furthermore, the UTAUT model was extended with trust and lifestyle compatibility factors to investigate smart wearable payment device adoption. The survey-based data were collected through the online survey and analyzed through the symmetrical modeling approach of partial least squares structural education modeling (PLS-SEM) to evaluate theoretical associations between the study constructs. The fuzzy set qualitative comparative analysis (fsQCA) was employed as an asymmetrical approach. As a result, it was found that the ease of use, lifestyle compatibility, and trust significantly impacted the intention to adopt smart wearable payment devices. However, social influence and facilitating conditions did not support the intention of adopting smart wearable payment devices. Adopting these devices requires policy and infrastructure development to harness the adoption of smart wearable payment devices. This paper is concluded with study limitations and future research suggestions.

## Introduction

Information and communication technologies (ICT) play a pivotal role in daily human life across the globe. Human health and sports activities are managed and facilitated using mobile devices that are linked to smartphones (Borowski-Beszta and Polasik, 2020). With the evolution of smartphones and consistent internet access, more outstanding benefits to the general public could be achieved (Humbani and Wiese, 2019). Smartwatches and other wearable mobile accessories initially emerged as mobile fashion equipment (Borowski-Beszta and Polasik, 2020). Smart wearable devices are popular among health enthusiasts and people with health issues who need to consistently monitor their body parameters to achieve a healthy life (Karjaluoto et al., 2020). A past study recorded a sharp increase in mobile payment upon the rise in FinTech uprising development (Aji et al., 2020; Bin, 2022). Although mobile payment acceptance is increasing, the subject of acceptance of smart wearable payment devices should be explored (GSMA, 2019; Baishya and Samalia, 2020).

Fintech and internet-based payment application development offer the convenience of executing monetary transactions while associated with security risks (Karjaluoto et al., 2020; Schinckus et al., 2021). The non-cash financial transactions require authentication and authorization to perform the payments (Lin et al., 2020). Cashless payment also offers a lifestyle, although it may not be compatible with many consumers (Karim et al., 2020; Bin, 2022). Moreover, many parties do not prioritize convenience and time-saving, while security remains the prime priority among consumers in developed and developing economies (Gumussoy et al., 2018; GSMA, 2019). However, trust and availability of prompt support could instigate the intention and adoption of cashless payment systems (Chawla and Joshi, 2019).

An increase takes place in smartwatch adoption worldwide, given that the global pandemic of COVID-19 has increased health concerns among people (Priem, 2021). Subsequently, the smartwatch is continuously a popular choice for monitoring health (Karjaluoto et al., 2020). Global manufacturers of smartwatches have begun offering advanced features in smartwatches and building near-field communication (NFC), which leads to the possibility of using smart wearable devices for payment features (NASDAQ, 2021). The contactless payment method also reduces the chances of being contracted COVID-19 and helps reduce the spreading of COVID-19 (Senyo and Osabutey, 2020; Birtus and Lăzăroiu, 2021).

The demand for smart wearable payment devices will increase in the coming years (NASDAQ, 2021). Smart wearable devices offer contactless payment for convenient shopping in retail and grocery stores while keeping a record of the payment transactions (Karjaluoto et al., 2020). However, using these devices does not reduce cash handling, while the fear of the spreading of COVID-19 enables instant payment of necessary bills (Senyo and Osabutey, 2020). Retailers are also installing contactless payment infrastructure and booths to keep their customers safe and reduce the waiting time and lines (GSMA, 2019; He and Li, 2020).

Bank Negara Malaysia (BNM) in Malaysia, which regulates the nation's financial industry, has issued 45 non-bank and six bank e-money licenses (Bank Negara Malaysia, 2020). The familiar platforms are Touch ‘n Go, Boost, GrabPay, Vcash, Razer Pay, Fave, KipplePay, and Air Asia's Big Pay. Furthermore, the Malaysian Government has encouraged the public to adopt cashless payment systems (Tariq, 2020). Simultaneously, cashless service providers actively promote cashless payment systems with cashback, coupons, and rebates (Singh et al., 2020; Rydell and Kucera, 2021). Meanwhile, the Malaysian government promotes these methods to encourage the public to use a cashless payment system, although only 8% of Malaysians use mobile wallets/cashless payment options (Nielsen Malaysia, 2019). The global wearable payment device market may involve an expenditure of US$82 billion by the end of 2026, with a 13.6% average annual growth (NASDAQ, 2021).

Wearable payment device is the future of finance (Andronie et al., 2021), which facilitates the execution of the disbursement of funds without any physical contact and builds a cashless society. Wearable payment devices are the technology that needs to be compatible with the lifestyle of the public and have the justified trust of the consumers. Wearable payment devices are the technology that facilitates the consumer and offers usefulness and ease of use (Andronie et al., 2021). The current work aims to discover the role of lifestyle compatibility and trust with other factors (perceived ease of use, perceived usefulness, social influence, and facilitating conditions) in adopting wearable payment devices. Moreover, the current study also explores the influence of wearable payment device attributes on the intention to adopt smart wearable payment devices with symmetric and asymmetric analysis techniques. fsQCA is the asymmetric technique that empowers the researcher to offer the possible configurations to understand the complex adoption behaviors (Hayat et al., 2022).

The next section offers the relevant literature and the development of the study hypotheses. Followed by the literature review, which reported on the method employed for the current study. The discussion and study conclusion are presented at the end of the section.

## Literature review

### Theoretical foundation

Since 2003, the unified Theory of Acceptance and Use of Technology (UTAUT) has been considered a robust theory to explore and understand technology adoption. The UTAUT advocated the existence of the power to demonstrate over 70% of the variance in the intention and adoption of technologies (Dwivedi et al., 2019). Furthermore, UTAUAT is extensively utilized to predict the technology adoption of smartphones (Baishya and Samalia, 2020), online learning (Chen and Hwang, 2019), social learning systems (Khechine et al., 2020), adoption of mobile payment (Gupta and Arora, 2019), adoption of Mobile commerce (Pandey and Chawla, 2019), and electronic payment wallet (Chawla and Joshi, 2019).

However, trust and compatibility issues were not included in the original UTAUT model, although trust and security are the essential attributes of technology adoption (Verma and Sinha, 2018). Smart wearable payment devices require higher perceived trust from consumers to perceive safety through smart wearable payment devices (Senyo and Osabutey, 2020). The trust reduces consumers' vulnerabilities and harnesses their confidence in using technology, including e-wallets or smart wearable payment devices (Gumussoy et al., 2018; Karim et al., 2020). Besides, the user would feel comfortable when the technology is compatible with the existing system or technology (Suki and Suki, 2017). Lifestyle compatibility creates perceived ease and confidence in using technology, such as smart wearable payment devices (He and Li, 2020).

The use of e-wallets among Malaysians relies primarily on promotional incentives, including discounts and rebates, prompting Malaysians to download mobile wallet applications without utilizing them (Nielsen Malaysia, 2019). Therefore, the encouragement of Malaysian users to use cashless payment devices for continuous and sustainable usage is a questionable subject. Preceding research works suggested that perceived usefulness and ease of use are considered essential factors for adopting technology, although other factors also affect the adoption of smart wearable payment devices.

### Hypotheses development

#### Perceived ease of use

Perceived ease of use denotes that technology is easy to use and the available instruction for the use of technology is clear and understandable (Dwivedi et al., 2019). The perception of ease of use for technology builds the provision of all the technology guidelines and the ease of interacting with technology (Baishya and Samalia, 2020). Consumer behavior is significantly influenced by the cutting-edge technology that can facilitate money transactions during COVID-19 (Priem, 2021). The e-payment systems bring ease of use for most consumers and help develop the positive intention to adopt the payment technologies (Chen and Hwang, 2019; Andronie et al., 2021). Based on the above pieces of evidence, the following hypothesis was proposed as follows:

Hypothesis (H_1_): *perceived ease of use of wearable payment devices positively affects intention to adopt the smart wearable devices*.

### Perceived usefulness

The awareness of consumers regarding mobile payment or cashless payment systems is increasing (Lin et al., 2020). The perception of usefulness describes the perception of benefits derived from the use of the technology (Chawla and Joshi, 2019). Perception of usefulness is based on the degree of the user's understanding that the use of technology brings benefits to the users, such as time-saving, state of satisfaction, and enhancement of performance in general. Using internet-based technologies help users bring efficiency to users (He and Li, 2020). Using Fintech offers time-saving and confidence to the technology users and harnesses the intention (Bin, 2022). Accordingly, the following hypothesis was developed in the current study:

Hypothesis (H_2_): *The perceived usefulness of wearable payment devices positively affects the intention to adopt smart wearable devices*.

### Perceived trust

The perception of trust depicts the personal understanding regarding the personal information that is not revealed to any individuals or security features that are present to protect the users' details (Slade et al., 2015). The perception of trust promotes the adoption of technology-related payment systems (Chawla and Joshi, 2019). The technology offers high-level security features to protect the users' information and transaction (Priem, 2021), which are made online to nurture the intention to use internet-based payment devices (Gu and Wei, 2020; Karim et al., 2020). Therefore, the following hypothesis was proposed in this study:

Hypothesis (H_3_): *The perceived trust in wearable payment devices positively affects the intention to adopt the smart wearable device*.

### Lifestyle compatibility

The perception of lifestyle compatibility is postulated as the consumers' perception that new technology is compatible with the consumers' existing needs, values, and general lifestyle (Chawla and Joshi, 2019). The general consumer attempts to reduce the efforts required to use the innovative technology, while high compatibility positively influences the intention to use the technology (Gumussoy et al., 2018). The users' habits and behavioral choices predict the purchase attitude and technology use behaviors (Rydell and Kucera, 2021). Lwoga and Lwoga (2017) postulated that the users' perception of compatibility promotes the intention to use mobile payment. Based on the previously highlighted discussion, the following hypothesis was formulated:

Hypothesis (H_4_): *Lifestyle compatibility of wearable payment device positively affect the intention to adopt the smart wearable device*.

### Social influence

Social influence is the perception of social acceptability measured by approval from the family members and peers within the individual's surroundings (Singh et al., 2020). Slade et al. (2015) stated that social influence promotes the positive intention toward using IoT for online payment. Acceptance of novel financial technologies requires social approval, while the adoption of internet-based technologies remains low (Dwivedi et al., 2019; Schinckus et al., 2021). The social influence builds the social acceptance for Fintech and harnesses the intention among new users (Andronie et al., 2021). The following hypothesis was proposed in this study:

Hypothesis (H_5_): *The social influence of wearable payment devices positively affects the intention to adopt the smart wearable device*.

### Facilitating conditions

The perception of facilitating conditions depicts the availability of support to accept and use the novel technologies (Giovanis et al., 2018). Facilitating conditions are associated with the availability of assistance, supervision, and support for the use of technology and after maintenance or after-sale service for the technology (Karjaluoto et al., 2020). Facilitating conditions simplify and simplify the use of technology, making it acceptable and suitable to potential users (Rydell and Kucera, 2021). The user develops a positive intention with the perception that the facilitating conditions are present (Dwivedi et al., 2019). We propose the following hypothesis:

Hypothesis (H_6_): *Facilitating conditions for wearable payment devices positively affect intention to adopt the smart wearable device*.

## Research methodology

### Research design

In this study, the quantitative method employed a deducted approach to explore the factors impacting the intention to adopt smart wearable payment devices among the study respondents in Malaysia. The data were collected in a cross-sectional manner for this explanatory research through the survey. The causal-predict data analysis technique, PLS-SEM, was utilized to test the study hypothesis. The asymmetrical analysis was performed with fsQCA.

### Population and sample

The target population of the current study comprised the young and educated working Malaysians. The sample size calculation was performed using G-Power 3.1 with a power of 0.95, effect size 0.15, and seven predictors. The required sample size amounted to 145 (Faul et al., 2007). Moreover, the minimum threshold of 200 samples was suggested for PLS-SEM (Hair et al., 2019). Given that the study aims to employ the second generation of statistical analysis technique of structural equation modeling, 500 respondents were collected. The snowball sampling technique utilized a few qualifying questions added to the survey and took the respondents' consent for participation in the study from the Malaysian respondents. The data collection was performed by posting the questionnaire on social media from January 2021 to March 2021. The respondents were asked to share the questionnaire link with their family, friends, and peers.

### Survey instrument

All the survey items for the study were adopted from previously validated and pretested survey instruments. Perceived usefulness was adapted from work by Chawla and Joshi (2019) and assessed with six items. The perceived ease of use for smart wearable payment devices was estimated with six items and the items adopted from work by Giovanis et al. (2018). Social influence was assessed with the five items taken from Slade et al. (2015) and Singh and Srivastava (2018). Furthermore, facilitating conditions are estimated with five items adapted from Giovanis et al. (2018) and Aji et al. (2020). The lifestyle compatibility of smart wearable payment devices was assessed with six questionnaire items adapted from Chawla and Joshi (2019). Following that, perceived trust was evaluated with five items adapted from Chawla and Joshi (2019) and Gu and Wei (2020). The intention to use smart wearable payment devices was gauged with six items adapted from Gupta and Arora (2019) and Gu and Wei (2020).

### Common method bias

Cross-sectional studies are commonly associated with common method bias, in which CMV issues are assessed using multiple methodological and statistical tools (Podsakoff et al., 2012). Harman's one-factor test was applied to determine the current study's CMV effect as a diagnostic technique.

The single factor accounted for 31.59%, which was below the recommended threshold of 40% in Harman's one-factor test that approved the inconsequential influence of CMV in this study (Podsakoff et al., 2012). The correlation of latent factors indicated that the issue of CMV was not present among the study constructs, with the correlation among the study latent constructs being lower than 0.90 (Podsakoff et al., 2012). The result is shown in Table 1. A full collinearity test was conducted to evaluate the CMV issue following Kock (2015) directions. The study variables regressed on the newly created outcome variable. The analysis outcome suggests that PEU (2.447), PUF (2.650), TRT (2.592), SIN (1.628), LCM (3.097), FCN (2.701), and INT (3.030) had the VIF score <3.3. The outcome proves that the CMV was not a critical issue for the current study.

**Table 1:** Latent constructs correlation.

	**PEU**	**PUF**	**TRT**	**SIN**	**LCM**	**FCN**	**INT**
PEU	1						
PUF	0.693	1					
TRT	0.619	0.640	1				
SIN	0.384	0.453	0.529	1			
LCM	0.652	0.730	0.729	0.543	1		
FCN	0.671	0.654	0.630	0.594	0.714	1	
INT	0.619	0.670	0.695	0.519	0.791	0.656	1

PEU, perceived ease of use; PUF, perceived usefulness; TRT, trust; SIN, social influence; LCM, lifestyle compatibility; FCN, facilitating conditions; INT, intention to use smart wearable devices.

**Source:** Author's data analysis.

### Multivariate normality

Hair et al. (2019) suggested evaluating the multivariate data normality before using SmartPLS. Multivariate normality for the study data was assessed with the Web Power online tool (source: https://webpower.psychstat.org/wiki/tools/index). Based on the calculated Mardia's multivariate *p*-value, the study data showed a non-normality issue, provided that the *p*-values were below 0.05 (Cain et al., 2017).

### Data analysis method

Following the multivariate non-normality in the study dataset, the study utilized partial least square-structural equation modeling (PLS-SEM). Hair et al. (2014) recommended that variance-based structural equation modeling is adopted to analyze this exploratory nature and non-normality issue study to present an in-depth explanation of variance in the structural equation model-dependent constructs.

SmartPLS 3.1 program was employed to analyze the data collected for the current study. Specifically, PLS-SEM is a multivariate exploratory method for analyzing the path structure of integrated latent constructs (Hair et al., 2019). Furthermore, it empowers researchers to show good working performance with non-normal data sets that has small data set. Essentially, PLS-SEM is a casual-predictive analytical tool to execute complex models with composites and no specific assumption of goodness-of-fit static requirements (Hair et al., 2014). PLS-SEM analysis is performed in two phases. Specifically, the first phase addresses model estimation, where the construct reliability and validity of the model construct are evaluated (Hair et al., 2019). Phase two is related to the evaluation of correlations of the models and systematic testing of the study path model (Hair et al., 2014). The study analysis performed with *r*^2^, *Q*^2^, and effect size *f*^2^ could explain the endogenous construct changes caused by the exogenous constructs (Hair et al., 2019).

PLSpredict is suggested by Shmueli et al. (2019) to confirm the model's critical endogenous construct and scrutinize prediction faults. The way of Q2 predict statistic appraises the predictive performance of the model for the authentication with the naïve yardstick designed by the PLSpredict method (Shmueli et al., 2019). PLSpredict approximates the naïve standard in the linear regression model (LM). A comparison between RMSE or MAE values for LM and PLS models confirms the illuminating supremacy of the two methods. Shmueli et al. (2019) advise that the PLS-SEM model lacks predictive power when the PLS-SEM model brings higher prediction errors than the LM standard. If most of the PLS-SEM analysis produces higher prediction errors than the LM benchmark, it depicts the little predictive power of the PLS-SEM model. If only a small number of PLS-SEM paths produces higher prediction errors than the LM benchmark, it specifies the medium power of the PLS-SEM model; if no indicator in the PLS-SEM model on more errors than the LM benchmark, the PLS-SEM model has higher predictive power (Shmueli et al., 2019).

The fuzzy set qualitative comparative analysis (fsQCA) asymmetric modeling technique is developed by combining fuzzy sets and fuzzy logic (Ragin, 2008). Asymmetric modeling manages the complexity as the regression-based modeling based on correlation and path values, which cannot fully capture the association between the variables due to non-linearity between the variables (Woolside, 2015). Furthermore, fsQCA could identify multiple possible solutions that lead to the same results, which are contrary to the regression analysis. In this case, only one possible solution offered leads to one result (Hayat et al., 2022). The high correlation among the variables refers to the collinearity issue and confounding variable, which does not receive proper control in the regression-based modeling approaches (Kaya et al., 2020). The asymmetric approach could manage the positive and negative logic, provided that the reliance on one-sided logic is misleading (Ragin, 2008). However, all the possible combinations that could lead to a positive outcome may not lead to a negative outcome (Hayat et al., 2022). The fsQCA facilitates the identification of sufficient and necessary conditions using Boolean algebra from the set of observations (Ragin, 2006).

Four-step processes were proposed for the use of the fsQCA. The original study variables included the five-point Likert scales rescaled from 0 to 1, where 0 refers to the full non-membership of the set, while 1 depicts the full membership (Ragin, 2008). This process is known as calibration (Hayat et al., 2022). The second step requires the necessity analysis, which is also labeled as configurational elements. A condition is deemed as necessary when its consistency score exceeds 0.90 (Ragin, 2000). Additionally, necessity analysis denotes the proportion of fuzzy set scores in all the cases, which are less than or equal to the corresponding scores in the study results (Hayat et al., 2022).

The third step of fsQCA analysis is conducted to achieve the trust table algorithm. In this case, the trust table produces the 2^∧*k*^ rows, where k shows the number of outcomes utilized in the analysis. In contrast, the row presents every possible combination among the causal conditions of the study (Ragin, 2008). For a sample of over 150, the outcome score of 3 is deemed acceptable (Kaya et al., 2020). In relation to this, consistency is identified as the degree to which cases correspond to the set-theoretic relationship expressed in a solution, while the threshold consistency is set at 0.75 (Ragin, 2006).


$$\begin{array}{l} \text{Consistency }({\text{CON}}_{\text{i}})\;=\;(\min({\text{CON}}_{\text{i}},{\text{ Y}}_{\text{i}}))/({\text{CON}}_{\text{i}}) \end{array}$$

The fsQCA analysis offers three solutions such as complex, parsimonious, and intermediate solutions (Ragin, 2008). Specifically, the complex solution offers the all-possible configurations or combinations of input variables that lead to an outcome. This solution is unnecessarily complex and impractical, given that the causal configuration is not possible (Hayat et al., 2022). The intermediate solution offers a mix of vital configurations that are parsimonious and complex (Ragin, 2008). The intermediate solution offers the core and peripheral conditions that can describe the possible outcome conditions removed in the parsimonious solution (Pappas and Woodside, 2021). Besides, the parsimonious solution only offers the vital configurations that lead to either easy or challenging outcomes (Ragin, 2006). Reporting intermediate solutions helps identify the core and peripheral conditions for achieving the outcome (Pappas and Woodside, 2021).

## Data analysis

### Demographic profile

As noted in Table 2, 51.6% of the total respondents were women. Most of the study respondents were represented by 45.5%, aged between 26 and 35 years old. Meanwhile, 39.2% of the respondents were between 18 and 25 years old, and 10.3% were aged between 36 and 45 years old. However, only 4.5% of the respondents were aged between 46 and 55. Following that, 4.6% of the respondents were between 31 and 35 years old, while 2.5% were between 36 and 40 years old. In terms of marital status, a majority of the respondents (60.9%) were single, while the remaining percentage comprised married individuals or divorcees. The respondents who completed their secondary school education were represented by 6.2%, while 10.7% of the respondents received a diploma or technical school education level. Moreover, 60% of the respondents received a college degree education. Regarding income, the respondents who received a monthly income below RM4000 amounted to 38.5%, while 27.5% received a monthly income between RM4001-RM8000. The remaining respondents received a monthly income above RM8001. Moreover, 69.3% of the respondents were working full time, while 13.4% were working part-time.

**Table 2:** Demographic characteristics.

	***N***	**%**		***N***	**%**
**Gender**			**Marital Status**		
Female	160	51.6	Single	189	60.9
Male	150	48.4	Married	113	36.4
Total	310	100.0	Divorced	6	2.0
			Widowed	2	0.8
**Age Group**			Total	310	100.0
18–25 years	121	39.2			
26–35 years	140	45.5	**Education**		
36–45 years	33	10.3	Secondary school certificate	20	6.2
46–55 years	14	4.5	Diploma certificate	33	10.7
56–65 years	2	0.5	Bachelor's Degree or equivalent	186	60.0
Total	310	100.0	Master's Degree	64	20.5
			Doctoral Degree	7	2.5
**Average Monthly Income (RM)**			Total	310	100.0
Below 4,000	119	38.5			
4,001–8,000	85	27.5	**Employment Status**		
8,001–12,000	40	7.9	Employed Full-Time	215	69.3
12,001–16,000	50	12.9	Employed Part-Time	42	13.4
16,001–20,000	10	2.0	Seeking opportunities	53	17.3
More than 20,000	6	1.3	Total	310	100.0
Total	310	100.0			

### Reliability and validity

Following Hair et al.'s (2019) suggestions, reliabilities for the study latent constructs were achieved and appraised through Cronbach's alpha (CA), DG rho, and composite reliability (CR). Cronbach's alpha values for each construct were above the threshold of 0.70, while the minimum value of Cronbach's alpha value amounted to 0.862 (Hair et al., 2014). The overall results are shown in Table 3. Similarly, all the DG rho values of the study constructs were above the threshold of 0.70, while the minimum value of DG rho was 0.865 (Hair et al., 2019).

**Table 3:** Reliability and validity.

**Variables**	**No. Items**	**CA**	**DG *rho***	**CR**	**AVE**	**VIF**
PEU	6	0.864	0.865	0.898	0.597	2.440
PUF	7	0.862	0.865	0.900	0.644	2.682
TRT	7	0.880	0.882	0.913	0.677	2.477
SIN	6	0.882	0.937	0.917	0.701	1.699
LCM	7	0.924	0.927	0.941	0.726	3.282
FCN	6	0.867	0.873	0.904	0.655	2.803
INT	6	0.918	0.925	0.936	0.710	-

PEU, Perceived ease of use; PUF, Perceived usefulness; TRT, Trust; SIN, Social influence; LCM, Lifestyle compatibility; FCN, Facilitating conditions; INT, Intention to use smart wearable devices; SD, Standard Deviation; CA, Cronbach's Alpha; DG rho, Dillon-Goldstein's rho; CR, Composite Reliability; AVE, Average Variance Extracted; VIF, Variance Inflation Factors.

**Source:** Author's data analysis.

Moreover, CR values were beyond the threshold of 0.70, where the lowest value of CR value was 0.898 (Hair et al., 2014). These results indicated that the latent constructs showed appropriate reliabilities and good performance for the subsequent analysis. The average value extracted (AVE) for all items for each construct should exceed the score of 0.50 to establish adequate convergent validity and support the uni-dimensionality concept for each construct (Hair et al., 2019). The items illustrated the adequate convergent validity for the constructs (see Table 3). All the value inflation factor (VIF) values for each construct were below the threshold of 3.3., indicating no multicollinearity concern (Hair et al., 2014). The item loading and cross-loading were reported for the confirmation of construct discriminant validity, as shown in Tables 3, 4. The study constructs showed fitting discriminant validities (see Table 4).

**Table 4:** Discriminant validities.

	**PEU**	**PUF**	**TRT**	**SIN**	**LCM**	**FCN**	**INT**
**Fornell-lracker criterion**							
PEU	0.773						
PUF	0.693	0.802					
TRT	0.619	0.640	0.823				
SIN	0.384	0.453	0.529	0.837			
LCM	0.652	0.730	0.729	0.543	0.852		
FCN	0.671	0.654	0.630	0.594	0.714	0.809	
INT	0.619	0.670	0.695	0.519	0.791	0.656	0.843
**HTMT ratios**							
PEU	-						
PUF	0.793	-					
TRT	0.713	0.728	-				
SIN	0.414	0.481	0.728	-			
LCM	0.723	0.808	0.807	0.574	-		
FCN	0.774	0.747	0.719	0.651	0.794	-	
INT	0.688	0.734	0.773	0.563	0.727	0.855	-

PEU, perceived ease of use; PUF, perceived usefulness; TRT, trust; SIN, social influence; LCM, lifestyle compatibility; FCN, facilitating conditions; INT, intention to use smart wearable devices.

**Source:** Author's data analysis.

The Fornell–Larcker criterion (1981) was utilized to achieve the discriminant validity for each study construct. This criterion was calculated with the square root of a particular construct AVE. Furthermore, the AVE square root for the construct should be higher than the correlation among the study's other constructs (Hair et al., 2019). Another suggested test for discriminant validity is the HTMT ratio, where the HTMT values should amount to 0.90 or less to establish the discriminant validity (Hair et al., 2019). Based on Table 4, the study showed no evidence for the lack of discriminant validity. Tables 4, 5 demonstrate that the study had sufficient discriminant validity for each construct.

**Table 5:** Loadings and cross-loading.

**Code**	**PEU**	**PUF**	**TRT**	**SIN**	**LCM**	**FCN**	**INT**
PEU1	0.776	0.518	0.484	0.289	0.484	0.556	0.443
PEU2	0.870	0.509	0.544	0.345	0.499	0.597	0.497
PEU3	0.756	0.462	0.495	0.319	0.461	0.546	0.418
PEU4	0.783	0.519	0.488	0.333	0.474	0.536	0.453
PEU5	0.694	0.556	0.421	0.239	0.528	0.386	0.523
PEU6	0.745	0.620	0.436	0.261	0.551	0.496	0.509
PUF2	0.543	0.836	0.508	0.374	0.591	0.520	0.509
PUF2	0.574	0.817	0.499	0.330	0.552	0.489	0.463
PUF3	0.535	0.793	0.449	0.250	0.503	0.487	0.484
PUF4	0.534	0.810	0.507	0.367	0.564	0.550	0.569
PUF5	0.581	0.753	0.579	0.458	0.682	0.554	0.623
TRT1	0.463	0.514	0.850	0.483	0.631	0.533	0.628
TRT2	0.512	0.535	0.848	0.450	0.611	0.524	0.564
TRT3	0.493	0.564	0.869	0.458	0.615	0.510	0.562
TRT4	0.507	0.454	0.778	0.358	0.554	0.468	0.538
TRT5	0.577	0.566	0.765	0.419	0.583	0.552	0.559
SIN1	0.354	0.454	0.458	0.908	0.506	0.550	0.456
SIN2	0.327	0.395	0.472	0.929	0.496	0.536	0.498
SIN3	0.421	0.463	0.516	0.903	0.534	0.601	0.491
SIN4	0.340	0.380	0.485	0.904	0.477	0.521	0.463
SIN5	0.042	0.071	0.217	0.431	0.127	0.125	0.160
LCM1	0.606	0.626	0.649	0.484	0.876	0.624	0.708
LCM2	0.559	0.610	0.635	0.493	0.885	0.637	0.703
LCM3	0.589	0.650	0.635	0.472	0.864	0.606	0.707
LCM4	0.591	0.630	0.648	0.475	0.861	0.657	0.677
LCM5	0.504	0.634	0.604	0.451	0.813	0.568	0.634
LCM6	0.470	0.580	0.549	0.391	0.809	0.551	0.606
FCN1	0.490	0.506	0.532	0.507	0.585	0.830	0.527
FCN2	0.533	0.465	0.437	0.469	0.573	0.813	0.503
FCN3	0.466	0.531	0.517	0.482	0.524	0.717	0.469
FCN4	0.635	0.621	0.570	0.461	0.648	0.828	0.608
FCN5	0.570	0.511	0.484	0.492	0.547	0.852	0.530
INT1	0.460	0.444	0.549	0.489	0.588	0.450	0.767
INT2	0.441	0.431	0.622	0.485	0.603	0.541	0.771
INT3	0.551	0.571	0.544	0.379	0.658	0.521	0.852
INT4	0.533	0.596	0.568	0.382	0.696	0.548	0.886
INT5	0.555	0.670	0.602	0.464	0.715	0.649	0.873
INT6	0.578	0.637	0.631	0.438	0.724	0.585	0.898

PEU, perceived ease of use; PUF, perceived usefulness; TRT, trust; SIN, social influence; LCM, lifestyle compatibility; FCN, facilitating conditions; INT, intention to use smart wearable devices; ADP, adoption of smart wearable payment devices.

The Italic values in the matrix above are the item loadings, and others are cross-loadings.

**Source:** Author's data analysis.

### Path analysis

Following the acceptable reliabilities and validities from the structural assessment of the study model, the following measurement assessment was employed to scrutinize the study hypothesis. The adjusted *r*^2^ value for the six exogenous constructs (i.e., perceived ease of use, perceived usefulness, trust, social influence, lifestyle compatibility, and facilitating conditions) on the intention to use the smart wearable payment devices represented 66.8% of the changes in the intention of using smart wearable payment devices. The predictive relevance (*Q*^2^) value for the part of the model amounted to 0.467, which demonstrated a large predictive relevance (Hair et al., 2014).

The model standardized path values, *t*-values, and significance levels are shown in Table 6. The path coefficient between PEU and INT (β = 0.074, *t* = 1.226, *p* = 0.110) indicated an insignificant but positive consequence of the perceived ease of use for smart wearable payment devices values on the intention to use smart wearable payment devices. The result formed no significant statistical support to accept the H1. The path value for the PUF and INT (β = 0.098, *t* = 1.699, *p* = 0.045) indicated that the perceived usefulness of the smart wearable payment devices impacts the intention to use the smart wearable payment devices, and the effect is statistically significant and positive. The path between TRT and INT (β = 0.174, *t* = 2.877, *p* = 0.002) illustrated the influence of trust in smart wearable payment devices on the intention to use smart wearable payment devices, which is positive and significant. The path coefficient for the SIN and INT (β = 0.064, *t* = 1.202, *p* = 0.115) represented a positive but insignificant effect, which presented the support for not accepting the argument that social influence affected the intention to use the smart wearable payment devices and offered no support for accepting the H4.

**Table 6:** Path coefficients.

**Hypotheses**		**Beta**	**CI - Min**	**CI – Max**	***T***	***P***	***r* ^2^**	***f^2^***	**Q^2^**	**Decision**
H_1_	PEU → INT	0.074	−0.019	0.181	1.226	0.110		0.007		Reject
H_2_	PUF → INT	0.098	−0.003	0.190	1.699	0.045		0.011		Accept
H_3_	TRT → INT	0.174	0.077	0.274	2.877	0.002		0.038		Accept
H_4_	SIN → INT	0.064	−0.023	0.153	1.202	0.115	0.675	0.007	0.467	Reject
H_5_	LCM → INT	0.465	0.309	0.586	5.599	0.000		0.203		Accept
H_6_	FCN → INT	0.062	−0.046	0.201	0831	0.203		0.004		Reject

PEU, perceived ease of use; PUF, perceived usefulness; TRT, trust; SIN, social influence; LCM, lifestyle compatibility; FCN, facilitating conditions; INT, intention to use smart wearable devices.

**Source:** Author's data analysis.

The path from LCM to INT (β = 0.465, *t* = 5.599, *p* = 0.000), which illustrated the influence of the local compatibility on the intention to use the smart wearable payment devices, was positive and significant. Therefore, the path presented the support to accept H5. Furthermore, the path between FCN and INT (β = 0.062, *t* = 0.831, *p* = 0.203) demonstrated the positive but insignificant influence of the facilitating conditions on the intention to use the smart wearable payment devices. Table 6 illustrates the path coefficients.

#### Predictive assessment

The assessment of predictive power of the current model was assessed. *Q*^2^ predict values for the intention to use smart wearable devices were above 0 and show the acceptable predictive power. The linear regression model (LM) and PLS-SEM model show that more of the LM benchmark yields more errors than the PLS-SEM model. The results deliver an acceptable indication that the PLS-SEM model performs well for the prediction purposes for out-of-sample results to predict intention to use smart wearable devices. The distribution of errors confirms that the intention to use smart wearable devices has high predictive power (see Table 7).

**Table 7:** Predictive model assessment.

	**Q^2^_Predict_**	**RMSE (PLS-SEM)**	**RMSE (LM)**	**Difference**	**Predictive power**
INT – Item 1	0.362	1.183	1.190	−0.007	
INT – Item 2	0.402	1.165	1.176	−0.011	
INT – Item 3	0.433	1.016	1.119	−0.103	High Predictive Power
INT – Item 4	0.471	0.938	0.992	−0.054	
INT – Item 5	0.539	0.948	1.001	−0.053	
INT – Item 6	0.541	0.896	0.947	−0.051	

INT, intention to use a smart wearable device; MAE, mean absolute error; RMSE, root mean squared error; PLS-SEM, partial least squares-structural equation modeling; LM, linear regression model.

**Source:** Author's data analysis.

### fsQCA results

The fsQCA analysis necessitated a calibration of the conventional variable gauged with the Likert scales. Each latent variable was estimated from the average values of the respective items in the latent constructs (Hayat et al., 2022). In the current study, six input conditions and one outcome were present. The seven variables of the study were calibrated into fuzzy sets using the direct calibration method. The threshold for full set membership was set at (fuzzy score = 0.95), while the non-membership threshold was set at (fuzzy score = 0.05), and the cross-over point was set at (fuzzy score = 0.50) (Kaya et al., 2020).

Table 8 illustrates the necessary condition analysis for the higher and lower intention to use the smart wearable payment devices. Specifically, higher intention demonstrated that no single casual condition achieved the consistency score of 0.90 (Ragin, 2008). Furthermore, the consistency score for each causal condition ranged from 0.873 to 0.770. It was clear that the casual combination of the condition could increase the intention to use smart wearable payment devices (Kaya et al., 2020). However, the lower intention to use smart wearable payment devices could also achieve the minimum consistency score.

**Table 8:** Necessary conditions for higher intention to use the smart wearable payment device.

	**High intention**		**Low intention**	
	**Consistency**	**Coverage**	**Consistency**	**Coverage**
PEU	0.873	0.873	0.536	0.488
PUF	0.817	0.828	0.545	0.490
TRT	0.770	0.757	0.605	0.562
SIN	0.835	0.836	0.558	0.508
LCM	0.815	0.813	0.569	0.520
FCN	0.776	0.787	0.608	0.545

PEU, perceived ease of use; PUF, perceived usefulness; TRT, trust; SIN, social influence; LCM, lifestyle compatibility; FCN, facilitating conditions; INT, intention to use smart wearable devices.

**Source:** Author's data analysis.

A truth table with a 2^k^ row was estimated. In the study with six casual conditions, the possible number of the combination was 64 (Ragin, 2008). The fsQCA analysis offered three solutions, with the intermediate solution representing the possible higher outcome achieved by combining casual conditions (Hayat et al., 2022). However, undesired combinations that reduced propositional consistency (PRI) were still present (Fiss et al., 2013). Table 9 illustrates the four casual paths, which led to a higher intention to use smart wearable devices. Overall, these paths showed a consistency value higher than 0.80, indicating an acceptable level of consistency (Ragin, 2008). The most acceptable and practical solution was known as the parsimonious solution (Fiss et al., 2013).

**Table 9:** Configuration of higher intention to use smart wearable payment devices.

**Configurations**	**Raw** **coverage**	**Unique** **coverage**	**Consistency**
PEU*PUF*FCN*trt*sin*lcm	0.275	0.027	0.965
PEU*LCM*TRT*puf*sin*fcn	0.297	0.034	0.942
PEU*PUF*SIN*LCM*FCN*trt	0.608	0.015	0.982
PEU*PUF*TRT*SIN*LCM*fcn	0.568	0.019	0.979
Total coverage		0.700	
Solution consistency		0.966	

PEU, Perceived ease of use; PUF, Perceived usefulness; TRT, Trust; SIN, Social influence; LCM, Lifestyle compatibility; FCN, Facilitating conditions; INT, Intention to use smart wearable devices.

Variable name in the capital shows the presence of a variable in causal combination (core conditions), the variable name in small caps shows the absence of variable (peripheral conditions).

“*”Present the combination of the factors that exist in the causal solution.

Furthermore, the first configuration demonstrated that the perceived ease of use, perceived usefulness, and facilitating conditions were adequate to achieve higher intention to use smart wearable devices despite the absence of trust, social influence, and lifestyle compatibility toward smart wearable payment devices. The first configuration covered 27.5% of the cases, uniquely describing 2.7% of the study cases with a higher intention to use smart wearable devices (Ragin, 2008). The second configuration indicated that the perceived ease of use, lifestyle compatibility, and trust were adequate to achieve higher intention to use smart wearable devices with the absence of perceived usefulness, social influence, and facilitating conditions toward intention to use smart wearable payment devices. Configuration 2 covered 29.7% of the cases and uniquely described 3.4%, indicating a higher intention to use smart wearable payment devices with acceptable consistency (Ragin, 2008).

The third configuration illustrated that the perceived ease of use, perceived usefulness, social influence, lifestyle compatibility, and facilitating conditions were suitable for achieving a higher intention to use smart wearable devices in the absence of trust toward smart wearable payment devices. The third configuration covered 60.8% of the cases and uniquely described 1.5% of the study cases for the higher intention to use the smart wearable devices, which presented a consistency score of higher than 0.80 (Ragin, 2008). The fourth configuration demonstrated that the perceived ease of use, perceived usefulness, trust, social influence, and facilitating conditions were adequate in achieving higher intention to adopt the smart wearable devices in the absence of facilitating conditions toward smart wearable payment devices. The configuration uniquely described 1.9% of the respondents' higher intention to use smart wearable devices (Ragin, 2008). The combination of four casual combinations could explain 70% of the higher intention to adopt smart wearable payment devices with acceptable consistency (Hayat et al., 2022).

The examination was performed on the causal asymmetry assumption based on fsQCA, which was a new absence of the higher intention to use smart wearable payment devices (Hayat et al., 2022). It presented the negation of higher intention to use the smart wearable payment devices within the study sample. As a result, the analysis demonstrated no significance for the four configurations initiated in the study. The result presented the causal asymmetry assumption relationship existing between the study variables.

## Discussions

The current study examined the formation of the intention to adopt smart wearable payment devices among Malaysian adults as an extension of the UTAUT with symmetric and asymmetric analyses. The study results supported the argument that the perceived ease of use, perceived usefulness, trust, and lifestyle compatibility influence the intention to use wearable smart payment devices. The ease of use and usefulness of the wearable smart payment device encourage young Malaysian professionals' intention to use smart wearable payment devices. The current study outcomes were in line with the result by Aji et al. (2020) that the perceived ease of use and usefulness of smart payment devices influenced consumers' intention to use the smart payment devices. Furthermore, trust influenced the study samples' intention to use smart wearable payment devices. The result of the current study was in line with the result presented by Slade et al. (2015). Trust plays an influential role in the adoption of Fintech, given that it helps consumers convert to new technologies and is considered less risky (He and Li, 2020).

However, social influence on the use of smart wearable payment devices was insignificantly negative in harnessing the intention to use smart wearable payment devices among the Malaysian samples. The young Malaysian respondents found that the important individuals around them did not favor using smart wearable payment devices. The result of the current study complemented the results by Gupta and Arora (2019), in which smart wearable devices were yet to show social acceptability. Nevertheless, the study result suggested that the facilitative conditions were not fully available to support the use of smart wearable payment devices. Overall, the findings of this study complemented the result reported by He and Li (2020), in which the Fintech consumer sought little help in using Fintech technologies. Malaysian respondents' lifestyle compatibility with smart wearable payment devices promoted the intention of using smart wearable payment devices. This study's result has supported the argument presented by Lwoga and Lwoga (2017), in which the perception of compatibility strengthened the consumer's intention to use technology. A comparable outcome was presented by Chawla and Joshi (2019), in which compatibility contributed to the intention to use mobile wallets among Indian consumers.

The fsQCA analysis offered a unique understanding regarding the higher intention to adopt smart wearable devices among the study cases. Four causal combinations of input conditions contributed to the higher intention for using smart wearable payment devices. The perceived ease of use and usefulness required facilitative conditions to promote higher intention toward using smart wearable payment devices. Similarly, localized compatibility was critical in building a higher intention to use smart wearable payment devices. Lastly, social influence and trust played a role in instigating higher intention to use the smart wearable payment devices, although it only took place in 2% of the cases. However, the four causal combinations of conditions covered ~70% of the cases, indicating the critical role the factors played in building a higher intention to use smart wearable payment devices.

### Theoretical implications

The current work made a theoretical contribution with the empirical evidence that the UTAUT model can be extended with trust and lifestyle compatibility factors. The perception of trust and lifestyle compatibility significantly influence the intention to use the smart wearable device as a technology. These two factors can enhance the UTAUT predictive power and acceptable addition of the UTAUT as a technology acceptance model. Furthermore, the current work supports that trust and lifestyle compatibility are important factors, indicating a higher intention to use the smart wearable payment devices with the asymmetrical data analysis technique of fsQCA.

### Policy and managerial implications

The current work has made attempts to contribute to the current technology adoption literature and managerial implications. Specifically, the positive inclination toward smart wearable devices shows positive signs for smart wearable device manufacturers. Subsequently, it was argued that more business opportunities are developing to expand the business, which is in line with the report by NASDAQ (2021). However, the adoption occurs at a slow pace, given that technology adoption takes time, although technology could gain the consumers' confidence. Similarly, the use of smart wearable devices for Fintech shows positive indicators, while the consumers in Malaysia gain less social support and facilitative conditions from financial institutions to fully adopt smart wearable payment devices. It is suggested that the banking institutions offer support and nudge to harness the adoption of smart wearable devices. Additionally, loyalty programs may contribute to higher adoption rates (Tariq, 2020).

Building localized compatibility assists in improving smart wearable payment device intention, followed by the adoption of smart wearable payment devices. The financial regulators and financial institutions should work closely with the local communities to build trust and social acceptance, and provide convenience to the less-educated individuals to use contactless payment devices. In the current time of the COVID-19 pandemic, it would be necessary to offer facilitative conditions through policy and monetary incentives to promote the use of smart wearable and contactless payment devices. This action also helps the reduction of personal contact and the spread of COVID-19.

Although a contactless payment system is the future of finance and banking, the right policy and managerial action should achieve higher penetration. Furthermore, the role of government support needs exploration, which contributes to understanding the role of facilitative conditions on the policy level that influences the adoption of smart wearable payment devices among the general public. In general, the availability of smart wearable payment devices, specifically contactless payment counters at shopping centers, facilitates the use of smart wearable payment devices. The mega and general store managers need to provide contactless payment devices to boost the adoption of smart wearable payment devices.

## Conclusion

There is an increase in the popularity of smart wearable devices for general health purposes and other relevant uses, while the acceptance of smart payment devices has increased over time (Karim et al., 2020). Smart wearable devices assist in incorporating everyday activities and discharge financial responsibilities smoothly and facilitatively. Contactless payment methods are necessary to reduce the spread of COVID-19 and assist the consumer and sellers. Furthermore, a current exploration was made on the intention to adopt the smart wearable payment devices using UTAUT, which was extended with lifestyle compatibility and trust factors. The survey-based data were collected and analyzed with symmetrical and asymmetrical analysis techniques. Essentially, ease of use, trust, and lifestyle compatibility positively influence the intention to adopt smart wearable payment devices. However, the asymmetrical analysis results demonstrated that consumers could develop a higher intention to adopt the smart wearable payment devices with the combination of ease of use, lifestyle compatibility, and facilitating conditions as casual conditions harnessing the intention to adopt the smart wearable payment devices.

Three prime limitations associated with the current study included using the UTAUT model. Therefore, future studies may be required to employ other adoption models based on the values and concern-based adoption. The value-based adoption model highlighted the implication of the derived value or cost associated with adopting novel technologies. Furthermore, government support and personal inclination toward technology are important in adopting technology. For this reason, future study is suggested to incorporate government support and individual personal readiness for technology adoption. The last limitation was the use of cross-sectional data collected in the current study. To fully explore the adoption of smart wearable payment devices, a longitudinal research design should be employed to understand the continuous intention and consistent use.

## Data availability statement

The original contributions presented in the study are included in the article/Supplementary material, further inquiries can be directed to the corresponding author.

## Ethics statement

Ethical review and approval was not required for the study on human participants in accordance with the local legislation and institutional requirements. The patients/participants provided their written informed consent to participate in this study.

## Author contributions

AS, MA, WH, and NZ: conceptualization, methodology, questionnaire design, data collection, and writing—original draft. NH and AA: conceptualization, formal analysis, and writing—reviewing and editing. All authors contributed to the article and approved the submitted version.

## Conflict of interest

The authors declare that the research was conducted in the absence of any commercial or financial relationships that could be construed as a potential conflict of interest.

## Publisher's note

All claims expressed in this article are solely those of the authors and do not necessarily represent those of their affiliated organizations, or those of the publisher, the editors and the reviewers. Any product that may be evaluated in this article, or claim that may be made by its manufacturer, is not guaranteed or endorsed by the publisher.
